# Numerical analysis of anisotropic wetting of chemically striped surfaces†

**DOI:** 10.1039/c8ra06626d

**Published:** 2018-09-12

**Authors:** Liang He, Xin Sui, Wenyan Liang, Zhenqing Wang, Abdolhamid Akbarzadeh

**Affiliations:** College of Aerospace and Civil Engineering, Harbin Engineering University Harbin 150001 China liangwenyan@hrbeu.edu.cn; Department of Bioresource Engineering, McGill University Island of Montreal QC H9X 3V9 Canada hamid.akbarzadeh@mcgill.ca

## Abstract

In this paper, the measurement process of advancing and receding contact angles (CA) in experiments is simulated using Surface Evolver (SE). The normalized energy of the droplet is calculated by fixing the three-phase contact line that lies at the boundary between stripes and by changing the droplet volume. The most stable wetting state is determined for each stripe configuration. The slip–jump behavior of the three-phase contact line is observed. Furthermore, a small wet stripe width and large dry stripe width is found to be favorable for achieving large stable equilibrium CA. Moreover, the minimum advancing CA and maximum receding CA can be obtained by assigning a value of zero to the normalized energy barrier. The variation of minimum advancing CA and maximum receding CA with wet and dry stripe widths follows the same trend as the stable equilibrium CA. Combined with the existing model in the literature, the approach introduced in this paper can be used to narrow down the predicted range of dynamic CAs and also to provide guidance for designing anisotropic surfaces.

## Abstract 2

The stabilities and dynamic wetting behavior of anisotropic wetting are investigated using surface evolver.

## Graphical abstract

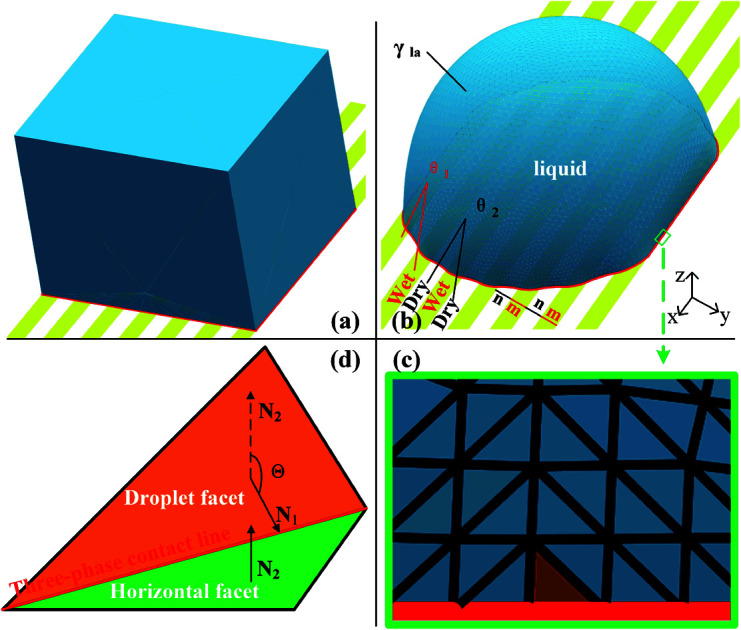

## 1. Introduction

Surfaces with unique wettability have received significant attention due to their potential applications, such as for self-cleaning,^1^ anti-icing,^2^ water transportation,^3^ and low-drag surfaces.^4^ Some of these applications involve anisotropy in wetting, where the directional motion of water can be controlled by tailoring the chemistry and roughness of surfaces. In addition, anisotropic surfaces are common in nature; for example, on rice leaves, microstructured papillae are arranged parallel to the rice leaf edge, and droplets can easily roll along a direction parallel to the rice leaf edge and pin along the perpendicular one;^5^ the anisotropic structures of butterfly wings enable droplets to slide off more easily along the radial outward direction than along the opposite one.^6^

Advances in micro/nanotechnologies have made it possible to design biologically-inspired anisotropic surfaces.^7,8^ Studies have shown that chemical and geometrical patterns are the two approaches to fabricate surfaces that exhibit anisotropic wetting.^7^ For example, chemically striped surfaces can be achieved by periodic assembly of alkylsilane,^9^ or perfluorosilane monolayers,^10^ or applying temperature gradients;^11^ geometrical patterned surfaces can be realized by photolithographic techniques,^8,12^ or femtosecond laser micromachining,^13^ or embossing and imprinting methods.^14,15^ Studies have shown that a droplet on an anisotropic surface shows an elongated shape,^16^ and the contact angle (CA) measured along the direction perpendicular to the stripes is larger than that measured along the direction parallel to the stripes. There is a general consensus that the three-phase contact line plays a dominant role in the wetting behavior of rough surfaces,^17–19^ and the strong anisotropy of droplets on striped surfaces is due to the contact line encountering discontinuity that causes the free energy (FE) barrier.^20^ While the static wetting behavior has been extensively investigated,^8,21–23^ the precise role of stripe properties in dynamic wetting behavior, such as advancing and receding CAs, is still not completely understood.

A number of experimental studies, typically focusing on the sliding behavior of anisotropic surfaces, have been conducted to understand the dynamic wetting. The sliding CAs along the directions parallel and perpendicular to the groove have been studied by Li *et al.*,^24^ and the result shows that the sliding CA measured along the direction perpendicular to the groove is much larger than that measured along the direction parallel to the groove. Another work reported by Wang *et al.*,^25^ concluded that the advancing CAs of anisotropic droplet increases with droplet velocity while the receding CA decreases with the velocity. In addition, a surface with programmable sliding direction is proposed by Zhang *et al.*,^26^ where a droplet can easily slide from the stretched end to the un-stretched end when the surface is stretched while cannot slide from the opposite direction. However, other parameters like advancing and receding CAs were not addressed. From the perspective of theoretical models, many thermodynamic models of anisotropic wetting have been proposed. One of these models is the one proposed by Li *et al.*,^27^ in which they established a 2D model by cutting the 3D grooved structures along a specific angle and calculated the CA and contact angle hysteresis (CAH) associated with FE and FE barrier. A similar 2D model based on FE minimization was presented by Chung *et al.*^16^ for the sinusoidal groove patterned surfaces, and the theoretical calculations were used to compare with experimental observation. In order to extend such a 2D model to a 3D one, He *et al.*^28^ used Surface Evolver (SE) to simulate the spreading process of a droplet on anisotropic surfaces, and studied the variations of droplet shape, CA, and dynamic CA (advancing and receding CAs). However, only the maximum CAH (maximum advancing CA and minimum receding CA) can be determined in the aforementioned 2D and 3D models.

As summarized above, it is of great interest to investigate the stabilities and dynamic wetting behavior of anisotropic wetting. In this paper, a methodology for predicting the advancing and receding CAs, as well as CAH is proposed. SE is used to study the wetting behavior of a droplet on anisotropic surfaces with features consisting of stripes with different wettability. The normalized form of free energy is used to investigate the relative stabilities of the droplets with different volumes for different stripe configurations. In addition, the process of experimental measurements of advancing and receding CAs are simulated by changing the droplet volume for each stripe configuration. The minimum advancing CA and the maximum receding CA can be obtained by assigning the zero value to the normalized energy.

## 2. Model calculation

Surface Evolver is a finite element-based software for studying surfaces confined by the energies such as surface tension, gravitational energy, and user-define surface integrals. The surface prescribed to a set of constraints in SE will be evolved towards its minimal energy using a gradient descent method. A detailed description of the technical procedure can be found in the SE manual.^29^

**Fig. 1:**
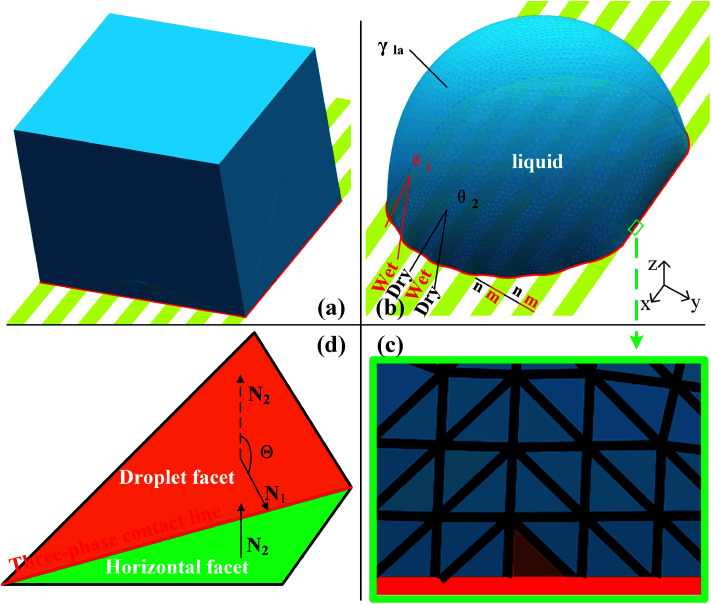
Simulation with surface evolver. (a) The initial droplet shape on top of 13 stripes. (b) The evolved droplet in the equilibrium wetting state. (c) Magnification of the region close to the contact line to illustrate a typical facet used for the contact angle measurement. (d) Schematic diagram of the contact angle measurement along the direction perpendicular to the stripes.

In this work, the wetting behavior of a droplet on chemically striped surfaces is simulated. The initial shape of the droplet in our simulation is defined as a cube, as shown in Fig. 1a. SE evolves the surface towards its minimum energy state (Fig. 1b) subjected to different constraints. Fig. 1b shows the stable state of the droplet with the volume of 0.72 residing on top of 13 stripes. As defined in Fig. 1b, the stripes in yellow have the intrinsic CA of *θ*_1_ and the width of *m*, and the white stripes represent those with intrinsic CA of *θ*_2_ and the width of *n*. In this study, *θ*_1_ is always smaller than *θ*_2_, and from now on, yellow and white stripes are referred to as wet and dry stripes, respectively. For the system of a droplet on chemically striped patterned surfaces, the total FE of the system calculated in SE is^23^1
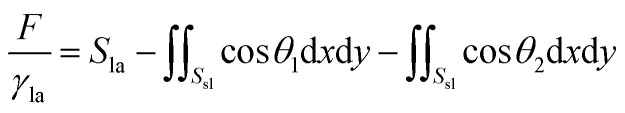
where *S*_la_ and *S*_sl_ represent the liquid–air and solid–liquid interface area, respectively; *γ*_la_ stands for the liquid–air interfacial tension. Here, FE is normalized by *γ*_la_, referred to as relative free energy hereafter. The intrinsic CA *θ*_1_ and *θ*_2_ of wet and dry stripes are defined by Young's equation:^30^

2 *γ*_la_ cos *θ*_*i*_ = *γ*_sa_ − *γ*_sl_

where *γ*_sa_ and *γ*_sl_ are the solid–air and solid–liquid interfacial tensions, respectively.

Gravity is ignored since droplet volume is kept sufficiently small for simulation to minimize the effect of gravity.^23,27,31^ Three-phase contact line tension is also neglected since the accumulated contact line energy is small enough compared to the total energy of the system. In SE, a surface is represented by the basic geometric elements including vertices, edges, and facets, as shown in Fig. 1c. Increasing the number of those geometric elements results in more accurate results of the simulation. In our case, the facets that come in contact with striped surfaces are deleted since those facets are not needed and may get in the way, as recommended by Brakke.^29^ The so-called “multiple one-sided constraint” is used to compensate the FE of the deleted facets using user-defined line integrals. In addition, other two kinds of constraints are used in simulation: the geometric constraint that ensures the constant droplet volume during the evolution process; and the constraint that confines vertices to stay in the corresponding boundaries between stripes. The values of 1, −cos *θ*_1_, and −cos *θ*_2_ are assigned to the liquid–air, wet stripe–liquid, and dry stripe–liquid interfaces according to eqn (1). The surface tension assignment is also shown in Fig. 1b. Assume that the normal of deleted the facet as ***S***, we need a vector field ***w*** that satisfies the following equation for line integral:3
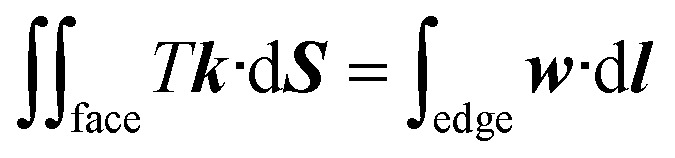
where *T* is the surface tension of the deleted facets; and it is −cos *θ*_1_ for wet stripes and −cos *θ*_2_ for dry stripes. Thus, either *w⃑* = −*Ty****i*** or *w⃑* = −*Ty****j*** can be selected for the line integrals along the three-phase contact line, where ***i***, ***j***, ***k*** are the unit basis vectors.

**Fig. 2:**
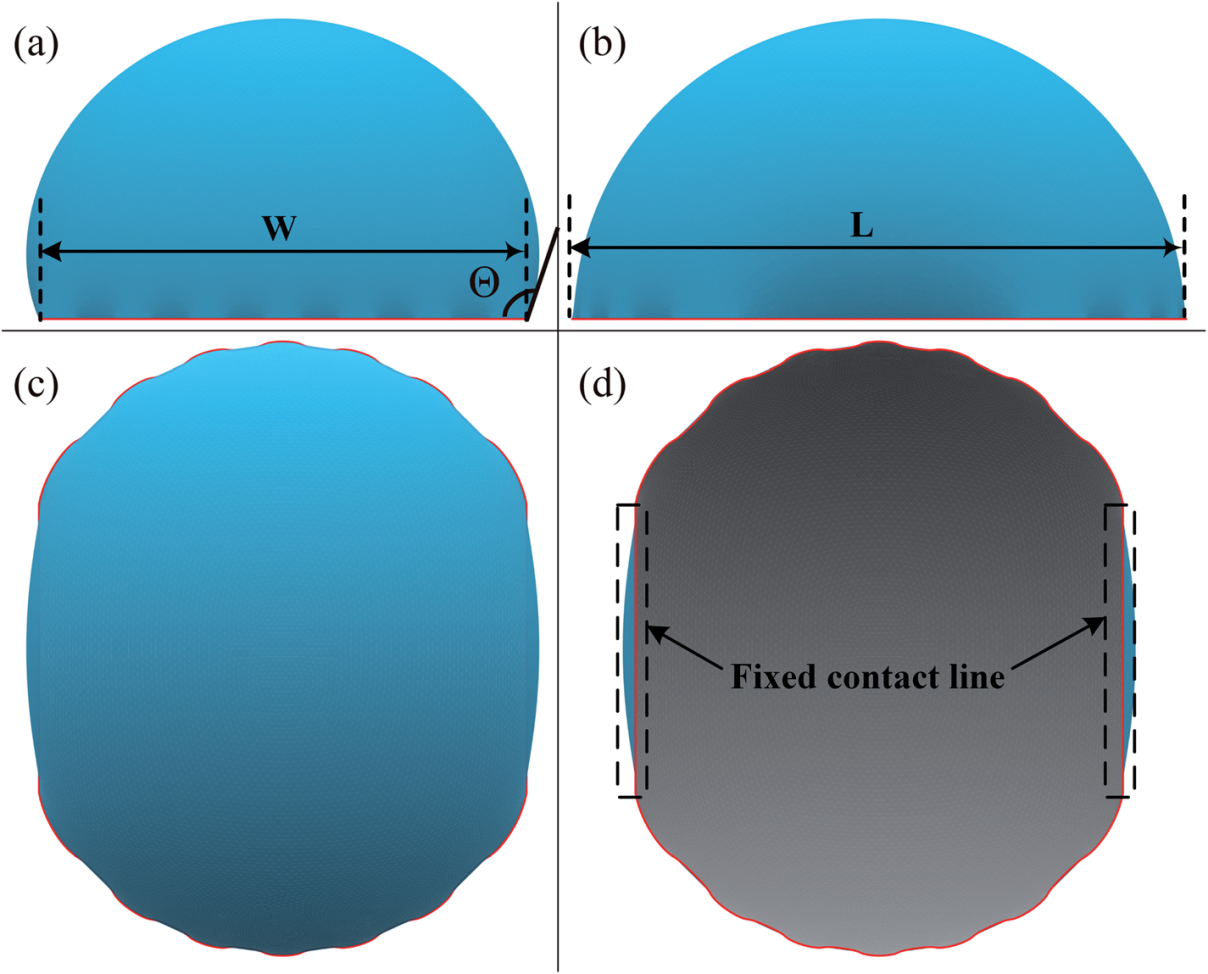
Shape of a droplet residing on top of 13 stripes, with the three-phase contact line highlighted in red. (a) Front view of the droplet shape, the definitions of the three-phase contact line width *W* and contact angle. (b) Side view of the droplet shape, and the definition of the three-phase contact line length *L*. (c) Top view of the droplet shape. (d) Bottom view of the droplet shape, and the fixed part of the three-phase contact line. The widths of dry and wet stripes are 0.1; the intrinsic contact angle for dry and wet stripes are 80° and 100°, respectively.

The focus of this work is the wetting behavior of the droplet along the direction perpendicular to the stripes, and the behavior along the direction parallel to the stripes is not discussed in the present study. The CA measured along the direction perpendicular to the stripes is defined in Fig. 2a. The measurement of CA in this work is depicted in Fig. 1d. Denoting ***N***_1_ as the normal vector of the facet (orange facet in Fig. 1c and d) that one edge of which is located at the boundary between stripes (the three-phase contact line), and ***N***_2_ as the normal vector of the horizontal surface (green facet in Fig. 1d), the CA can be calculated using dot product:4
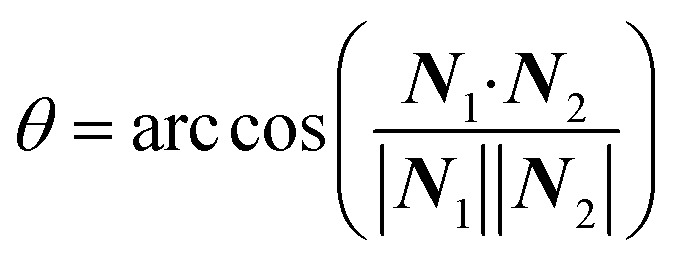


The CA is determined by calculating the average value of all legitimate facets. Grid independence is also checked to obtain the reliable results of the simulation. In brief, grid independence is considered achieved when the change of converged energy is less than 10^−6^ after each successive refinement, and the facet number of droplet surface should be greater than 20 000. The experimental study of Jansen *et al.*^32^ has also been used to validate the simulation procedure by comparing the aspect ratio and the CA for a droplet on chemically striped surfaces, detailed information can be found (ESI†).

## 3. Results and discussions

A typical run of a droplet with the volume of 0.72 residing on top of 13 stripes is shown in Fig. 2 with *m* = *n* = 0.1, *θ*_1_ and *θ*_2_ are 80° and 100°, respectively. All the parameters in simulation are nondimensionalized since SE only deals with numerical values. The three-phase contact line width *W* and length *L* are respectively defined in Fig. 2a and b to characterize droplet distortion behavior. The three-phase contact line is highlighted in red. As seen from the Fig. 2, an elongated shape of a droplet on striped patterned surfaces is observed. The three-phase contact line exhibits a wave-like shape where the convex and concave parts are located on wet and dry stripes, respectively. This prevents that the contact lines on dry stripes from being visible from the top view, as shown in Fig. 2c. It should be noted that the droplet with the volume of 0.72 may not be in the most stable wetting state with the configuration in Fig. 2, the stability of a droplet using the method proposed by Chatain *et al.*^31^ They suggested that the stabilities of drops can be investigated by fixing the three-phase contact line and changing drop volume, and normalized energy is a key parameter that describes the droplet relative stabilities, and can be written as:5
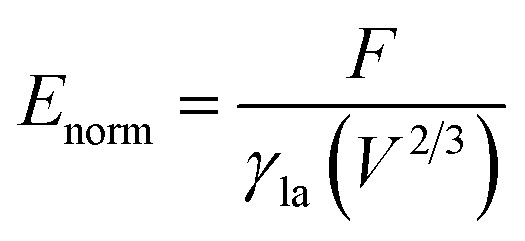
where *V* is the droplet volume. For a free sessile droplet on ideally smooth horizon surfaces, *E*_norm_ is a constant value and a function of intrinsic CA, droplet volume does not play a role here.^33^ However, if the three-phase contact line of a droplet is fixed, *E*_norm_ will exhibit variety values for different droplet volumes. Hereafter, *E*_norm_ is used to investigate the wetting stabilities and hysteresis phenomenon of a droplet residing on different number of stripes as its volume is increased or decreased.

**Fig. 3:**
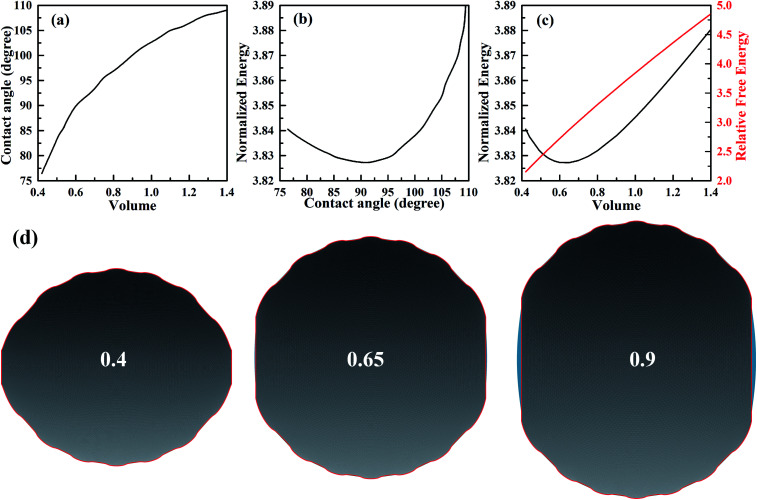
(a) The variation of contact angle as a function of the droplet volume. (b) The variation of normalized energy as a function of contact angle. (c) The variations of normalized energy (black line) and relative free energy (red line) as a function of droplet volume. (d) The bottom view of a series of droplets of different volumes residing on top of 13 stripes. The volume of each drop is given in the centre of each picture. The widths of dry and wet stripes are 0.1; the intrinsic CAs for wet and dry stripes are 80° and 100°, respectively.

In order to investigate the wetting property of striped patterned surface along the direction parallel to stripes, the portion of three-phase contact line that lies on the boundaries between stripes is fixed (Fig. 2d) and the rest that lies on stripes is unfixed. Fig. 3 presents the relationships among CA, droplet volume, normalized energy, and relative FE of a droplet residing on top of 13 stripes. The intrinsic CAs for wet and dry stripes are 80° and 100°, respectively. As seen from Fig. 3a, CA shows an increasing trend as droplet volume increases because of the constrained contact line. However, normalized energy decreases first and then increases with CA, and the minimum normalized energy near a CA of 91.66° is the stable equilibrium contact angle. This minimum value of normalized energy correspond to the most stable wetting state. In addition, both normalized energy and relative FE are plotted as a function of droplet volume in Fig. 3c. The relative FE shows almost a linear relation with droplet volume; however, the normalized energy first decrease with the volume and reaches to its minimum at the volume around 0.65. For droplet volumes other than 0.65, an additional elastic energy will be introduced because the contact line at the boundaries between stripes is fixed, and those droplets are in metastable wetting state.^31^ The increasing of droplet volume also causes an increase in the length of three-phase contact line. The shapes of a series of droplets with different volumes residing on top of 13 stripes from bottom view are presented in Fig. 3d. The volume is given in the centre of each droplet. Because the contact line that lies on stripes is not fixed, droplet spreads along the stripe direction to minimize the total energy. As the volume increases from 0.4 to 0.65, the contact line length increases from 1.12 to 1.37. It is worthy to mention that the contact line length of 1.37 of the droplet in most stable state is very close to the contact line width of 1.30. This finding that for the stripe arrangement in Fig. 3, the contact line width and length become more and more closer to each other as the droplet approaches to its stable state, is identical to the result of our previous study on spreading behavior of droplet on striped patterned surfaces by keeping droplet volume unchanged.^28^

**Fig. 4:**
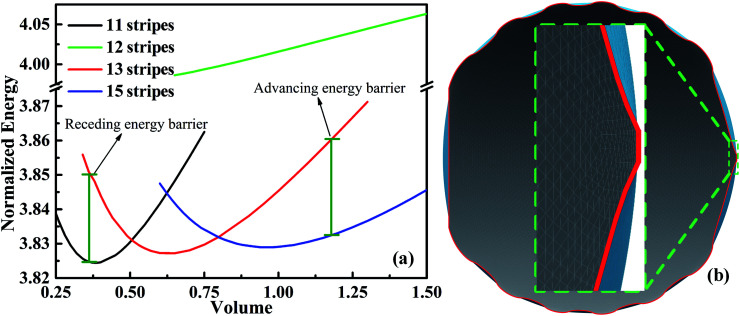
(a) The variation of normalized energy of droplets of different configurations on top of stripes. (b) The bottom view of the droplet with the volume of 0.625 for 12-stripe configuration. The widths of dry and wet stripes are 0.1; the intrinsic CAs for wet and dry stripes are 80° and 100°, respectively.

Next we consider an experimental dynamic CA measurement by adding/removing liquid.^34^ For measuring the advancing CA, this method involves placing droplets onto solid surfaces and then gradually increasing droplet volume. At first, the CA increases while the three-phase contact line is constant. With the increasing of volume, a three-phase contact line begins to move forward when volume reaches a certain value. The CA measured at this moment is defined as advancing CA. Similarly, the CA measured before contact line starts to move backward as a result of withdrawing liquid from droplets, is known as the receding CA. Based on this methodology, we can use SE to simulate the dynamic CA measurement by calculating the stabilities of droplets on different positions with respect to different stripe numbers. We have calculated several scenarios of droplets on top of 11, 12, 13, and 15 stripes. As shown in Fig. 4a, the normalized energy for volume near the minimum value of each configuration is calculated. The outer two stripes that droplet occupies for 11-, 13-, and 15-stripe configurations are wet stripes.^35^ The normalized energy of 12-stripes configuration is much larger than that of other three configurations. This indicates that 12-stripes configuration is always unstable compared with the 11-, 13-, and 15-stripe configurations. Bottom view of the shape of droplet residing on top of 12 stripes with the volume of 0.625 is presented in Fig. 4b. The inset shows the droplet shape on the twelfth stripe. It is clear that the three-phase contact line shrinks inward to the eleventh stripe. SE returns the result that is not physical and difficult to convergence when the droplet volume is smaller than 0.625. The above discussion has indicated that the droplet will move directly from 11-stripe configuration to 13-stripe configuration as the volume increasing.

**Fig. 5:**
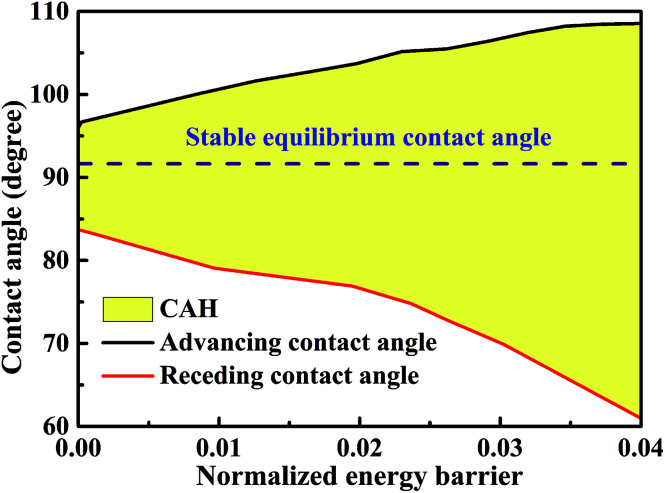
The variation of advancing and receding contact angles for the 13-stripe configuration as a function of normalized energy barrier. The stable equilibrium contact angle of the droplet in the stable wetting state is shown in blue dash line. The widths of dry and wet stripes are 0.1; the intrinsic CAs for wet and dry stripes are 80° and 100°, respectively.

The minimum normalized energy for each droplet volume forms a minimum envelope of the curves in Fig. 4a. There is an intersection of each two curves, droplets with the two configurations are stable at the same time. Continue of increase (decrease) droplet volume may cause the three-phase contact line moves outward (inward) to the next wet stripes, and then the droplet changes its configuration. Whether the three-phase contact line moves or stays depends on the energy barrier that droplet need to overcome form one configuration to another.^36^ A droplet needs an addition energy to overcome the energy barrier. This addition energy may be realized by changing droplet volume, vibrating, pressure, and other methods.^37^ This energy barrier is the reason for the occurrence of the CAH which is defined as the difference of advancing and receding CAs. The values of advancing and receding CAs depend on the value of energy barrier. In this study, the advancing and receding CAs can be obtained if the normalized energy barrier is known, as shown in Fig. 4a. The variation of advancing and receding CAs for the droplet on top of 13 stripes in Fig. 4 are presented in Fig. 5 as a function of the normalized energy barrier that droplet needs to overcome to change its configuration. The blue line shows the equilibrium CA of droplets in most stable wetting state. The advancing CA increases while receding CA decreases as the normalized energy increase. The receding CA shows larger deviation from stable equilibrium CA than advancing CA as the normalized energy barrier increases. As a consequence, a large normalized energy will result in a large CAH. Furthermore, if we consider the normalized energy barrier as having the value of zero, the advancing and receding CAs can be obtained as 96.68° and 84.23°, respectively. The resulting CAH is determined to be 96.68° − 84.23° = 12.45°. It should be noted that the methodology by assuming a zero of normalized energy barrier results in the minimum advancing CA, maximum receding CA, and minimum CAH. This methodology helps us to investigate the dynamically anisotropic wetting behavior of a droplet on striped surfaces and narrow down the predicted ranges of advancing and receding CA, as well as CAH.

**Fig. 6:**
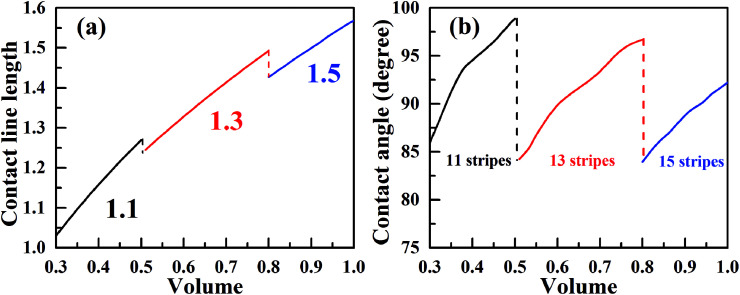
The slip–jump behavior of the three-phase contact line of a droplet on the striped surfaces with *m* = *n* = 0.1, *θ*_1_ = 80°, and *θ*_2_ = 100°; the normalized energy barrier is assumed to be zero. (a) The variation of contact line length as a function of droplet volume, the three-phase contact line width for each configuration is given in the same color as the curve. (b) The variation of contact angle as a function of droplet volume.

Now, we consider the droplet volume increasing process for the surface in Fig. 4 with the normalized energy barrier of zero. When the volume of a droplet with 11-stripe configuration increases to near 0.5, both 11- and 13-stripe configurations are simultaneously stable. Continuing of increasing volume will cause the transition of droplet from 11- to 13-stripe configuration. The transition from 11- to 12-stripe configuration cannot proceed because the required normalized energy for this transition is too large. Similarly, droplet will transit from 13- directly to 15-stripe configuration at the volume around 0.8. The transition from 11- to 15-stripe configuration involves the changes in not only the contact line width (from 1.1 to 1.5), but also the contact line length. The variation of contact line length as a function of the droplet volume with zero normalized energy barrier is depicted in Fig. 6a. The contact line width is given in the same color as the corresponding curve. As seen, the contact line length increases with increasing of droplet volume for all the three configurations. When the volume reaches to the transition value, both contact line width and length jump outward to their next position. This slip–jump behavior is also associate with CA change. The variation of contact angle as a function of droplet volume with zero normalized energy barrier is shown in Fig. 6b. Taking 13-stripe configuration as an example, the maximum and minimum CAs correspond to advancing and receding CAs are 96.68° and 84.23°, respectively. Based on the abovementioned discussion and the CA measurement procedure, all the values between 96.68° and 84.23° are possible for the experimental static CA measurement as long as the volume used in experiment lies in the range from 0.5 to 0.8. We also notice that the curves in Fig. 6a are nearly parallel to each other, indicating that the contact line length increase linearly as the volume increases. In addition, for the scenarios in Fig. 6a, the contact line length shows a slight decrease when the droplet transit from one configuration to another. This slip–jump behavior has also been found by Jansen *et al.*^35^ They studied the droplet shapes deposited on a chemically striped patterned surfaces consisting of alternating hydrophobic and hydrophilic stripes, and found that the aspect ratio^22^ increased linearly first as the scaled ratio (volume) increases, and the aspect ratio drops to the value below unity after reaches its maximum value, following this the droplet transit to the next wetting state. The same transition is observed in Fig. 6a. Taking the transition from 13- to 15-stripe configuration as an example, the contact line length increases lineally with droplet volume while the contact line width remains 1.3; when contact line length reaches the maximum value, the droplet transit to next wetting state and the contact line width jumps to 1.5 while the contact line length jumps to the value smaller than 1.5 (the aspect ratio below the unity). The slip–jump behavior of contact angle (in the perpendicular direction) was also observed by Jansen *et al.*,^35,38^ they found that the droplet grown in the direction parallel to the stripes and the contact angle increases as droplet volume increases; when the contact angle reaches the maximum value, the droplet moves to the next wetting state leading to a decrease in contact angle. This finding is also observed in Fig. 6b. Here, we should point out that the methodology proposed in this paper is based on the ideal of normalized energy^31^ to analyze the wetting stabilities and the hysteresis phenomenon of anisotropic wetting, and is different from the work of Jansen *et al.*^35,38^ in which the focus was on the relationship between surface parameters and droplet shapes.

**Fig. 7:**
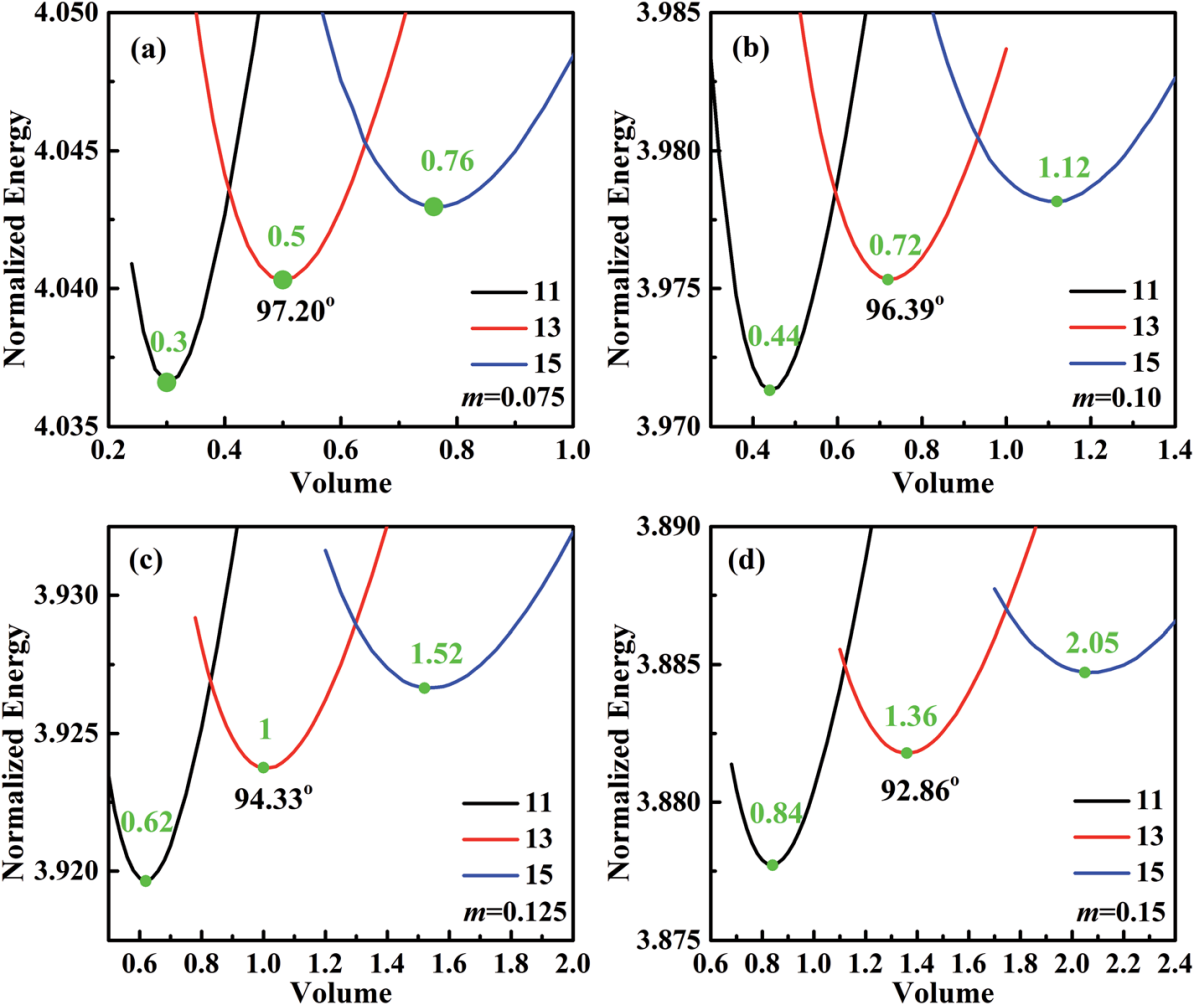
Normalized energy of droplets of 11-, 13-, and 15-stripe configurations on the striped surfaces. The dry stripe width is fixed to be 0.1, and the wet stripe widths are (a) 0.075, (b) 0.1, (c) 0.125, and (d) 0.15. The intrinsic CAs for wet and dry stripes are 80° and 110°, respectively.

**Fig. 8:**
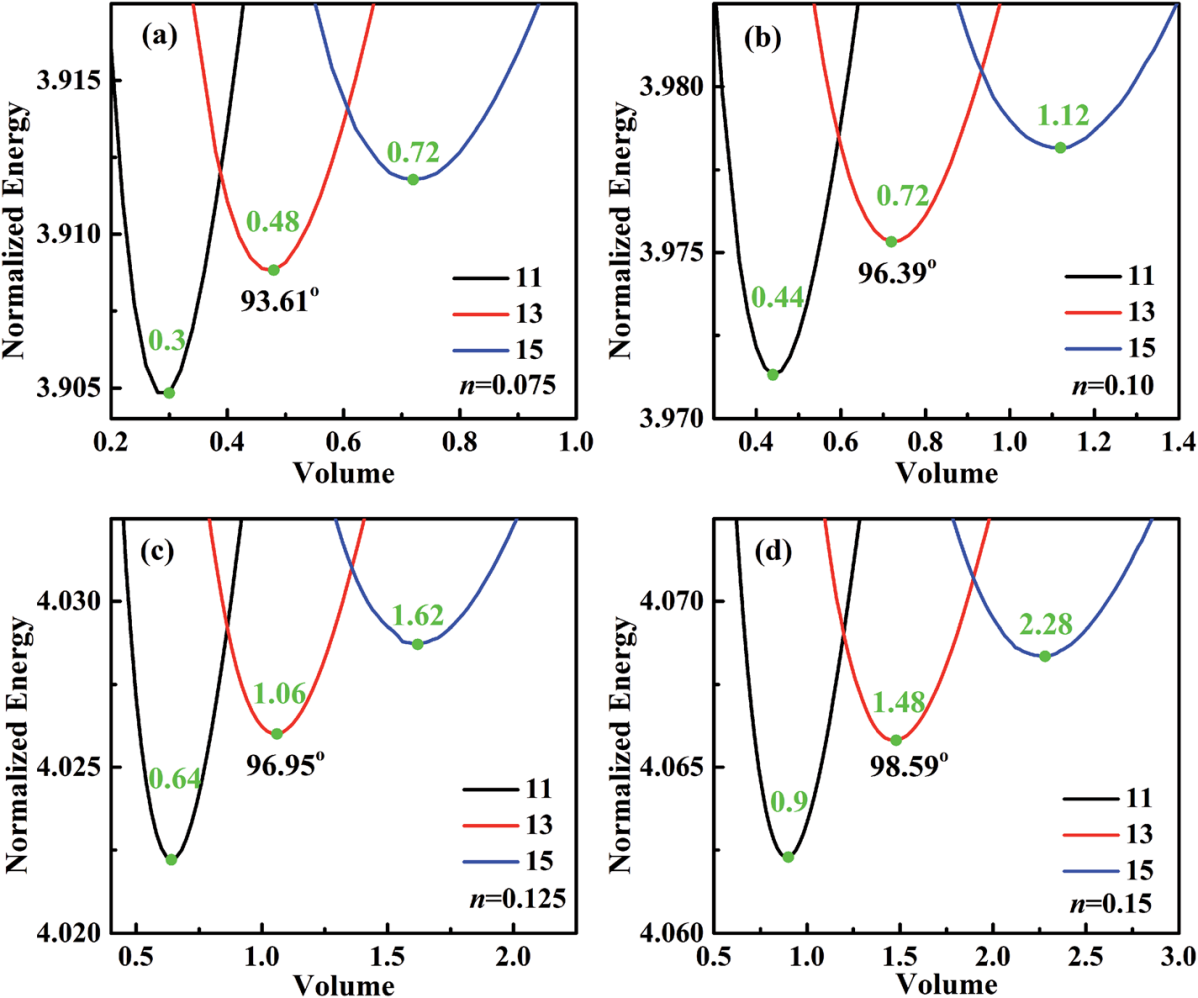
Normalized energy of droplets of 11-, 13-, and 15-stripe configurations on the striped surfaces. The wet stripe width is fixed to be 0.1, and the dry stripe widths are (a) 0.075, (b) 0.1, (c) 0.125, and (d) 0.15. The intrinsic CAs for wet and dry stripes are 80° and 110°, respectively.

For analyzing the effect of stripe width on wetting behaviors and stabilities, we have calculated the normalized energy of droplets on solid surfaces with respect to different stripe widths. The effect of wet and dry stripe widths on normalized energy are shown in Fig. 7 and 8, respectively. The dry (wet) stripe width is kept to be 0.1 while wet (dry) stripe width varies from 0.075 to 0.15 in Fig. 7 (Fig. 8). The intrinsic CAs for wet and dry stripes are 80° and 110°, respectively. Each curve shows a minimum value, and as discussed before, this minima of normalized energy corresponds to the most sable wetting state of that stripe configuration. The volume droplet in stable wetting state for each stripe-configuration is also given in the Fig. 7. The normalized energy is calculated for the volumes around the minimum value for each configuration. The most stable wetting state of each configuration is indicated by a green dot. It appears from Fig. 7 and 8 that the normalized energy increases with the increasing of stripe number that droplet occupies. This increasing trend is observed from all the considered cases. From the perspective of stability, the system is becoming more stable as the droplet volume and stripe number decrease. In addition, the stable equilibrium CAs are also given for each 13-stripe arrangement. As shown in Fig. 7 where *n* is fixed to be 0.1, the equilibrium CA drops from 97.20° to 92.86° as *m* increases from 0.075 to 0.15; while for the stripe arrangement in Fig. 8 where *m* is fixed to be 0.1, the equilibrium CA increases from 93.61° to 98.59° as *n* increases from 0.075 to 0.15. This indicates that small *m* and large *n* are favorable for achieving large stable equilibrium CA. Moreover, normalized energy of the most stable state decreases with the increase of *m*, as shown in Fig. 7; while the opposite effect of *n* on normalized energy of the most stable state is observed, as shown in Fig. 8. For the 13-stripe configuration, the normalized energy of stable state decreases from 4.04 for *m* = 0.075 to 3.88 for *m* = 0.15 (Fig. 7); while it increases from 3.91 for *n* = 0.075 to 4.07 for *n* = 0.15 (Fig. 8). This means that the system is more stable as *m* increases and *n* decreases according to the principle of minimum energy. Finally, based on the aforementioned methodology of determining CAH, the effects of wet and dry stripe widths on advancing, receding CAs, and CAH can also be obtained from Fig. 7 and 8.

**Fig. 9:**
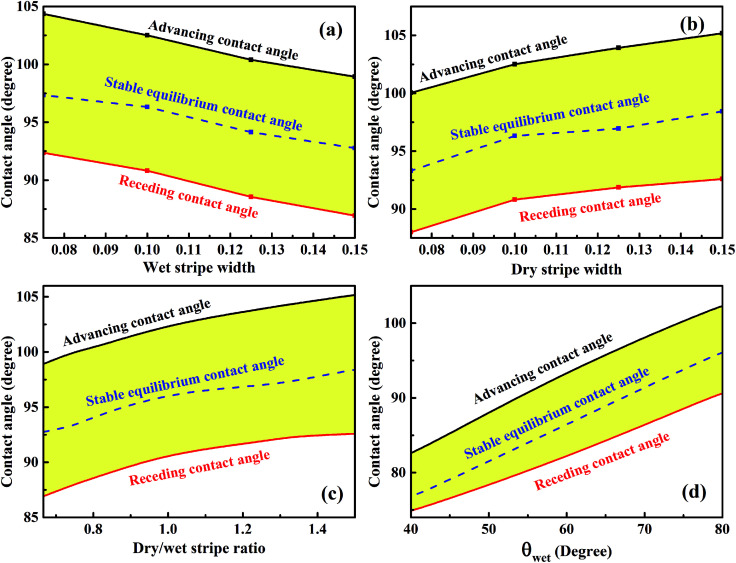
The variations of advancing and receding contact angles for the droplet of 13-configuration as a function of (a) wet stripe width, (b) dry stripe width, (c) dry/wet stripe width, and (d) intrinsic contact angle of wet stripes.

The variations of minimum advancing CA and maximum receding CA as a function of wet and dry stripe widths for the droplet residing on top of 13 stripes obtained from Fig. 7 and 8 are shown in Fig. 9a and b. The normalized energy barrier is assumed to be zero. The stable equilibrium CA is highlighted in blue. In Fig. 9a, both minimum advancing and maximum receding CAs decreases as the increase of *m*. This finding is in good agreement with the experimental results.^39^ In ref. 39, Shi *et al.* fabricated a series of micro-channel structures, both of the advancing and receding CAs decrease with the increasing of pillar width (*m*). In Fig. 9b, it is observed that the increasing of *n* causes the increases in both minimum advancing and maximum receding CAs. For both of the two cases, the resultant minimum CAH remains nearly constant, and the minimum advancing and maximum receding CAs show the same variation tendency as the stable equilibrium CA of the most stable wetting state. From the perspective of dry/wet strip ratio, as shown in Fig. 9c, the minimum advancing and maximum receding CAs, as well as the stable equilibrium CA exhibit the same tendency and increase as the dry/wet stripe width. In addition, the variation of the wettability of wet stripe as a function of CAs is plotted in Fig. 9d to investigate the effect of difference in wettability between the stripes on the wetting behavior. The wet and dry stripe widths are fixed at 0.1, and the intrinsic CA of wet stripes ranges from 40° to 80° while that of dry stripes is fixed at 110°. It is clear that the stable equilibrium CA increases as the intrinsic CA of wet stripes, as a result, the corresponding minimum advancing and maximum receding CAs also exhibit the same tendency. It is worth mentioning that there is also a method for determining the maximum advancing CA and minimum receding CA by assuming a zero value of relative free energy barrier.^27,28,40,41^ The main idea proposed in ref. 27, 28, 40 and 41 is keeping droplet volume constant and calculating the FE and FE barrier associated with microstructures for the droplet in all possible wetting states. Here, the approach that varies the droplet volume and calculates normalized energy for all possible wetting configurations narrows down the range of theoretical prediction of dynamic wetting. The approach in the present paper is a necessary complement for the scientific community of anisotropic wetting.

## 4. Conclusion

The wetting behavior of a droplet residing on anisotropic surfaces consisting of chemically-striped patterned surfaces is investigated by numerical methods using SE software. The normalized energy is used to characterize the wetting stabilities and dynamic wetting behavior of anisotropic droplets. The three-phase contact line along the boundary between stripes is fixed and the droplet volume is changing for each stripe configuration. The wetting state where the droplet resides on top of even number of stripes is found to be unstable, and the contact line will advance or recede to the next configuration with odd number of stripes. The three-phase contact line is found to advance or recede through a slip–jump mechanism similar to experimental observations; both width and length of the three-phase contact lines increase (decrease) as the contact line advances (recedes) from one configuration to another one, which is different from the spreading behavior of the anisotropic droplet.^28^ Furthermore, a small wet stripe width and large dry stripe width is found to be favorable for achieving large stable equilibrium CA. In addition, the minimum advancing CA, the maximum receding CA, and the minimum CAH are obtained by assuming a zero value of the normalized energy barrier. The minimum advancing CA and the maximum receding CA decrease (increase) with the wet (dry) stripe width, and exhibit the same variation trend as the stable equilibrium CA of the droplet in the most stable wetting state. The approach presented in this paper does not only provide insight into the anisotropic wetting but also narrows the predicted range of advancing and receding CAs combined with the available thermodynamic models in the literature.^27,28^

## Conflicts of interest

Authors declare that there is no conflict of interest.

## Supplementary Material

RA-008-C8RA06626D-s001
