# Supplementary material for: Numerical analysis of anisotropic wetting of chemically striped surfaces
Source: RSC Adv. 2018 Sep 12;8(55):31735–44. doi: 10.1039/c8ra06626d (PMC9085812; doi:10.1039/c8ra06626d)
Supplement: RA-008-C8RA06626D-s001 [file RA-008-C8RA06626D-s001.pdf]

Electronic Supplementary Material (ESI) for RSC Advances.
This journal is © The Royal Society of Chemistry 2018

Electronic supplementary information

Numerical Analysis of Anisotropic Wetting of Chemically Striped Surfaces

Liang He [a, b], Xin Sui [a], Wenyan Liang [a*], Zhenqing Wang [a], Abdolhamid Akbarzadeh [b*]

[a] College of Aerospace and Civil Engineering, Harbin Engineering University, Harbin 150001, China

[b] Department of Bioresource Engineering, McGill University, Island of Montreal, QC H9X 3V9, Canada

[*] *Corresponding authors: Wenyan Liang (Email: liangwenyan@hrbeu.edu.cn) and Abdolhamid Akbarzadeh (Email: hamid.akbarzadeh@mcgill.ca)*

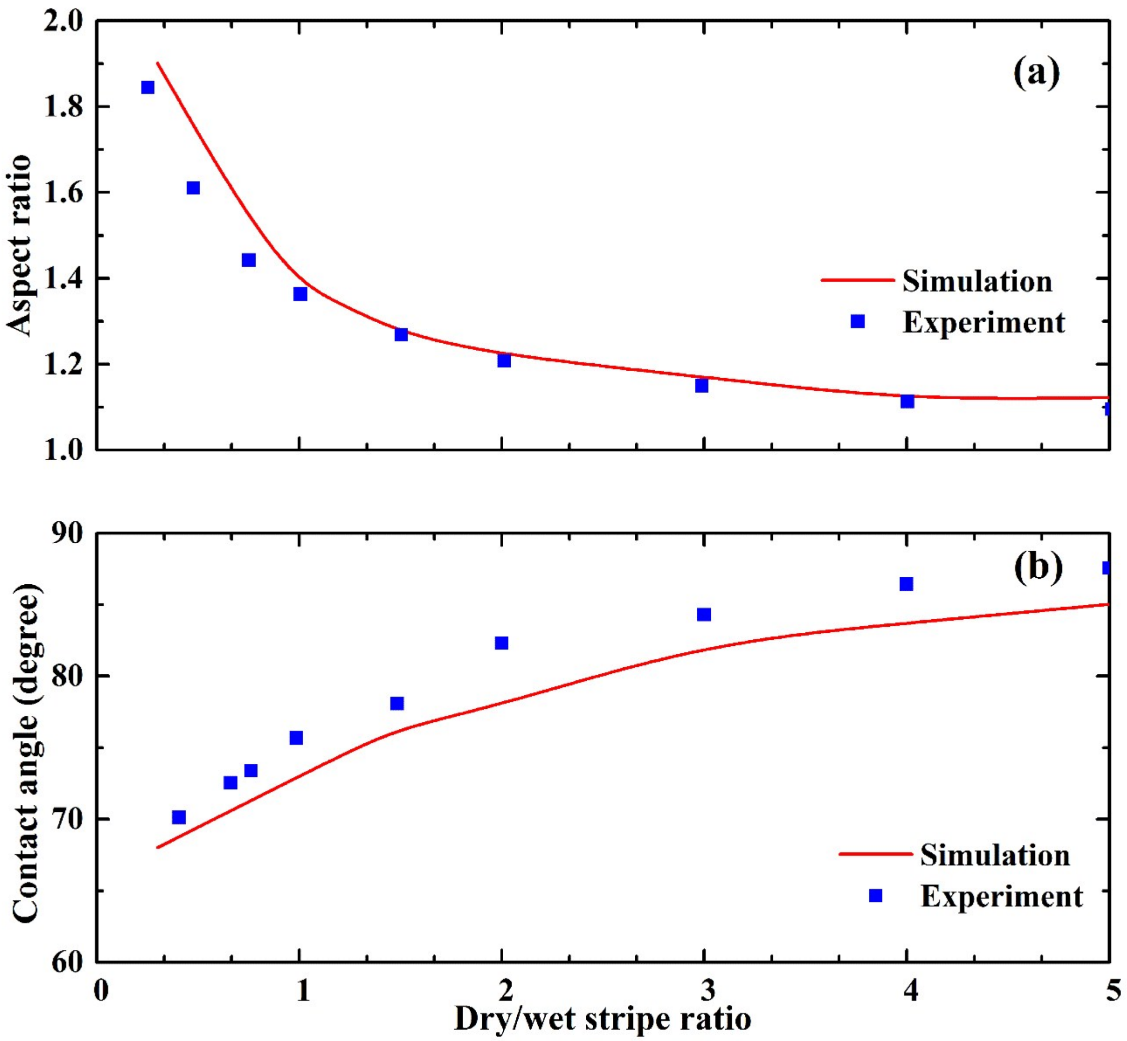

Fig. S1 (a) Aspect ratio of the droplet plotted as a function of dry/wet stripe ratio from experiments (blue squares) and simulation (red line).The intrinsic contact angles 96° (dry) and 19° (wet) are used for the simulation. (b) Contact angle plotted as a function of dry/wet stripe ratio from experiments (blue squares) and simulation (red line).

The work of Jansen et al. [1] is used for validating the numerical simulation procedure. They fabricated a set of surfaces consisting of alternating hydrophobic and hydrophilic stripes using self-assembled perfluordecyltrichlorosilane monolayers on oxide-coated silicon wafers. Simulations are done with the intrinsic contact angles of 96° and 19° for dry (PFDTS) and wet (SiO_2) stripes, respectively. The validation process involves (i) obtaining an elongated shape similar to the experimental results, and (ii) using the contact angles obtained from simulation to compare with the experimental observations. The experimental data used in the comparison is from the data reported in Figure 1 of the reference. As seen from Fig. S1, the experimental result are in agreement with the simulated data. There are slight deviations between experimental and simulated data, the difference may be caused by the different measurement procedures of the contact angle.

Another simulation work by Jansen et al. [2] also shows the similar slight deviations from the experimental observations since a small difference in aspect ratio results in observable change in contact angle. However, the overall trend of simulation results agrees well with the experimentally measurements, indicating the validation of the proposed methodology in the present paper.

References:

1. H. P. Jansen, K. Sotthewes, C. Ganser, C. Teichert, H. J. Zandvliet and E. S. Kooij, *Langmuir*, 2012, **28**, 13137-13142.
2. H. P. Jansen, O. Bliznyuk, E. S. Kooij, B. Poelsema and H. J. Zandvliet, *Langmuir*, 2012, **28**, 499-505.
